# Absconding from a psychiatric hospital in Johannesburg, South Africa: Are we seeing a decrease since the implementation of the *Mental Healthcare Act*?

**DOI:** 10.4102/sajpsychiatry.v25i0.1338

**Published:** 2019-12-04

**Authors:** Feroza Arbee, Ugasvaree Subramaney

**Affiliations:** 1 AKESO Clinic, Parktown, Johannesburg, South Africa; 2 Department of Psychiatry, School of Clinical Medicine, Faculty of Health Sciences, University of the Witwatersrand, Johannesburg, South Africa

**Keywords:** abscond, absconding rate, absconder, escape, psychiatric facilities

## Abstract

**Background:**

Absconding from psychiatric facilities, the aetiology and impact of which have major socio-economic implications, has a multifactorial aetiological basis. Absconding patients are at higher risk of self-harm, violence, non-adherence, relapses, substance use and negative media attention. Most health professionals associate absconding with the escape of potentially dangerous psychiatric patients. Absconding causes fear and uncertainty, and portrays psychiatric services negatively. Identification of potential absconders would assist with risk assessment and prevention.

**Aim:**

The aim of this study was to formulate an absconding rate as well as a descriptive profile of absconders since the inception of democracy and deinstitutionalisation.

**Setting:**

The study was conducted at Sterkfontein Hospital, a specialised psychiatric hospital outside Johannesburg.

**Methods:**

A retrospective record review of absconders from Sterkfontein Hospital in Johannesburg over 1 year was conducted.

**Results:**

The absconding rate was 7.83%. The characteristics of the typical absconder included single, unemployed male, early 30s, known to psychiatric services, diagnosed with schizophrenia and co-morbid substance use. An absconder is more likely to be a forensic patient not returning from official leave of absence.

**Conclusion:**

The absconding rate has decreased to less than half that of a previous study, and is within international norms. While the descriptive profile is of limited value, it does appear that psychiatric patients are being treated in a less restrictive manner resulting in fewer absconders and a change in the method of absconding. The implications for clinical practice are firstly that a clearer definition of the term absconding is needed as this will impact risk assessment and management. It is recommended that future studies separate forensic and general populations. Lastly, the formulation and use of a risk assessment tool may be of value.

## Introduction

A standard definition of absconding remains elusive, making comparison between studies difficult.^1^ According to Bowers et al.,^2^ the most popular definition seems to be that of Antebi^3^ – ‘leaving the hospital grounds without permission, or failing to return from leave’. The consequences of absconding include harm to self or others, non-adherence, relapses, violent behaviour,^4^ substance abuse, intensive nursing, poorer prognosis and a negative portrayal of psychiatric services.^5^

Meehan et al.^6^ postulate that the potential absconder is a young single male schizophrenic who comes from a disadvantaged background. However, these patients are more likely to be admitted to inpatient psychiatric services^7,8^ leading to an over-representation of these characteristics. Absconding rates vary widely (2.5% – 34% of all psychiatric patients) because of the different definitions^5^ and calculations used.^4^

Pages et al.^9^ suggest that if potential absconders could be identified, interventions could be implemented to ensure that these patients complete treatment, rather than repeatedly using inpatient services for crisis management. Some of the reasons for absconding include boredom, lack of visitors, relationship problems, impulsivity, non-compliance, peer influence, active symptomatology (e.g. command hallucinations), efforts to obtain substances, dislike of staff, ward or food, feeling that their admission is unnecessary,^6^ feeling confined and denial of leave or discharge.^5^

In 1994, South Africa inherited the apartheid legislation of outdated and highly institutionalised psychiatric services. With democracy and the accompanying *Mental Health Care Act* No. 17 of 2002 (MHCA),^10^ there have been parallel moves to address the problems of stigmatisation, discrimination, prolonged institutionalisation, inaccessibility to appropriate care and exploitation of mentally ill patients. The MHCA is considered to be among the most progressive mental health legislations in the world.^11^ Unfortunately, deinstitutionalisation has not been resoundingly successful. Locally, service integration has been hampered by infrastructure constraints, administrative challenges and limited political support.^11,12,13^ Kaliski^13^ points out that deinstitutionalisation, with its enforced psychiatric bed reduction and increased turnover of patients, has resulted in a paradoxical increase in the number of involuntary admissions. Thus, the population at risk of absconding is potentially larger.

There have been two previous studies on absconding in South Africa.^14,15^ Both of these studies had findings in keeping with international findings. However, these studies are outdated and to our knowledge, no new data have been published.

## Aims

The aims were to calculate an absconding rate, formulate a descriptive profile of patients who absconded and compare data with that of a study carried out at the same hospital prior to the change in mental health legislation and deinstitutionalisation.

## Methods

The study was conducted at Sterkfontein Hospital, a specialised psychiatric hospital outside Johannesburg. It is affiliated to the University of the Witwatersrand’s Department of Psychiatry and is thus a teaching hospital. It treats both general (for further involuntary care under the MHCA) and forensic psychiatric patients.

Awaiting-trial detainees are admitted for a 30-day observation period under the *Criminal Procedure Act* (CPA).^16^ If, following this period, they are found not to be fit and/or not criminally responsible by virtue of mental illness or defect, and the crime was minor, they can be referred for general admission under the MHCA as involuntary patients. If the crime was major/serious, they are admitted to the forensic unit as state patients.

For this research study, all patients who left the hospital premises without being discharged were considered absconders. This includes patients on leave of absence (LOA) who do not return timeously, as well as escaped patients (those who run away or find a way out of the hospital without being given LOA/discharge).

## Study design

The study was a retrospective record review of all psychiatric patients who absconded from Sterkfontein Hospital in 2008. Approval was obtained from the Human Research and Ethics Committee and the hospital Clinical Executive Officer (CEO). Access to patient files was restricted to the principle researcher.

## Sample

Data on all documented absconding cases were obtained from daily incident reports, forensic administration statistics, ward movement books and patient files. All wards comprising Sterkfontein Hospital were investigated (i.e. both general and forensic wards). Where there were missing data in the file of an absconder, this was excluded from the analysis.

## Data and/or tools

The data collection sheet included age, gender, relationship and employment status, section of the MHCA or CPA under which admitted, general or forensic ward, open or closed ward, psychiatric diagnoses, substance use, factors that may have contributed to absconding, presence of past psychiatric history, documented previous admissions, date of admission, date of absconding, method of absconding, previous absconding incidents, length of hospital stay and whether returned or not.

## Absconding rate

The absconding rate was calculated using the widely accepted formula by Molnar and Pinchoff:^17^

Na/Npr X 100 [Eqn 1]

Where Na = number of patients absconding and Npr = number of patients at risk of absconding (i.e. the total number of inpatients at the beginning of the study period plus patients admitted during the course of the study).

When calculating the event-based absconding rate, repeat absconding incidents by the same patients are included, resulting in higher rates. The patient-based absconding rate takes into account the number of patients who have absconded, regardless of how many times they absconded.

## Data analysis

Descriptive statistical and associational (Chi-square and Fisher’s exact tests) analyses were used to summarise the data and provide a measure of variability. The data were analysed using the Statistica 9.1 and Stata 11.0 programs. If more than one absconding incident was recorded for a patient, it was analysed separately. For the associational analysis, only the first absconding incident was considered for repeat absconders. As a large proportion of the sample was made up of patients not returning from LOA and the risk assessment and management of these patients differ substantially from escaped patients, these were differentiated in the statistical analysis.

### Ethical considerations

This article followed all ethical standards for a research without direct contact with human or animal subjects.

## Results

A total of 97 patients that absconded during 2008 fulfilled criteria for the study. Seven patients absconded more than once, resulting in a total of 108 absconding incidents that were analysed. The event-based absconding rate was 7.83% and the patient-based absconding rate was 7.03%.

## Socio-demographic profile

Table 1 reflects the socio-demographic characteristics of the patients.

**TABLE 1:** Socio-demographic characteristics of patients.

Variable	*n*	%
**Age (year)**		
≤ 18	3	3.09
19–25	19	19.59
26–35	34	35.05
36–45	25	25.77
46–55	13	13.40
≥ 56	3	3.09
**Gender**		
Male	82	84.54
Female	15	15.46
**Employment status**		
Unemployed	80	82.47
Receiving disability grant	10	10.30
Employed	7	7.22
**Relationship status**		
Single	83	85.57
Married	10	10.31
Divorced/separated	2	2.06
Widowed	2	2.06

The mean age of the combined absconding group was 33.8 years. The mean age of the escape group was 26.2 years. The association between young adulthood (age 19–25) and escape was statistically significant (*P* < 0.001). Male patients accounted for 84.54% (*n* = 82) of all absconding patients while 15.46% (*n* = 15) were female patients. The escape sample consisted of 100% male patients (*n* = 22) and there was a statistically significant link between male gender and escaping (*P* = 0.02). No association was found between employment or relationship status and the method of absconding.

## Method of absconding and other variables

In 80 (74.07%) of the absconding incidents, the patient did not return from an official LOA. The remaining 28 (25.93%) incidents comprised patients escaping while under direct hospital supervision. Methods of escape included breaking ward doors or windows, through the roof, unlocked ward doors, while on ground parole (i.e. occupational therapy [OT], sports events, church and tuck-shop visits) and jumping the fence. Others escaped while receiving medical consultations or procedures at neighbouring district hospitals.

Thirty-one patients (31.96%) absconded from the general wards while under involuntary care and 68.04% (*n* = 66) patients who absconded were forensic state patients. If only escaped patients were considered, then 63.64% were general involuntary patients and 31.82% forensic state patients. Chi-square analysis revealed that involuntarily admitted patients were more likely to escape than state patients (*P* < 0.001), while state patients were more likely to delay their return from LOA as their primary method of absconding.

There was a statistically significant relationship between general wards and escapes (*P* < 0.001); however, the patients in the open wards were not more likely to escape (*P* = 0.32). The mean length of hospital stay was 1531 days (i.e. 51 months). Almost three quarters (71.43%, *n* = 70) of patients had been admitted for longer than 6 months. There was a statistically significant link between patients admitted for less than 6 months and escaping (*P* < 0.001).

**FIGURE 1:**
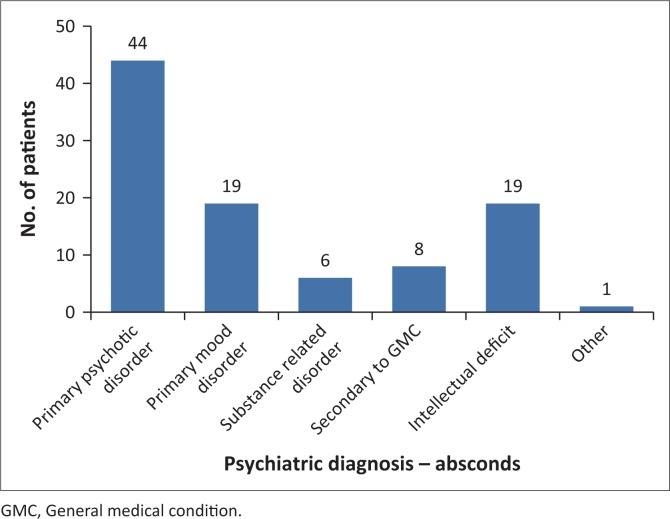
Distribution of psychiatric diagnoses (according to the diagnostic statistical manual of disorders, text revised (DSM IV TR) across sample population.

Of the 97 patients who absconded, 44 (45.36%) were diagnosed with a primary psychotic disorder (see Figure 1). Second in prevalence were two diagnoses with 19 (19.59%) each: Primary Mood Disorder and Intellectual Deficit. Fisher’s exact test did not elicit a statistically significant link between any particular psychiatric diagnosis and method of absconding.

Three quarters of the sample (74.23%) used substances (most commonly cannabis and alcohol), 24.74% (*n* = 24) denied using any substances and in 1.03% (*n* = 1) of patients substance use was not clear. Twenty-three patients (23.71%) had contributing factors documented in their files prior to absconding, while for 76.29% (*n* = 74) there were none. In the escape population, the frequency of documented contributing factors increased to 40.91% (9 out of 22). This link was statistically significant (*P* = 0.03).

Repeat absconders were those who absconded more than once during the study period (Table 2). Of the 108 absconding incidents, 18 (16.67%) were by patients who had absconded repeatedly in 2008. One patient escaped four times during the study period. There were no associational relationships found between past psychiatric histories, month of absconding, previous absconding incidents, patients returned or re-admitted and other variables.

**TABLE 2:** Repeat absconders.

Number of absconding incidents	Absconding patients	% of absconders (*n* = 97)	% of repeat absconders (*n* = 7)
1	90	92.78	-
2	4	4.12	57.14
3	2	2.06	28.57
4	1	1.03	14.29

## Discussion

The event-based absconding rate was 7.83%, while the patient-based absconding rate was 7.03%. According to a literature review by Bowers et al.,^2^ the mean rate of absconding for general psychiatric services is 12.6 with a range of 2–44. If patients not returning from official LOA were excluded from the definition used, the absconding rate would be even lower. In 1993 (with data recorded by Siwinska),^15^ the absconding rate was 16.70%. Both Karani and Siwinska’s reports did not utilise the formula of Moltar and Pinchoff in calculating the absconding rate for their studies. Notwithstanding this, this research study indicated that the absconding rate was less *than half* that of Siwinska’s study. This reduction may be partially accounted for by retrospective bias (i.e. under-reporting, missing files and incomplete data). The rest reflects a genuine reduction in the number of absconding incidents. The implementation of the MHCA No. 17 of 2002^10^ has resulted in the reduction of psychiatric beds, premature discharges, inadequate community-based resources with subsequent relapse and re-admission of patients involuntarily.^13^ This high turnover results in earlier discharges and LOA, reducing the opportunity to abscond.

Conversely, the forensic mental health system has seen a burgeoning in the number of state patients since the advent of deinstitutionalisation – aptly named ‘Reinstitutionalization by stealth’ by Kaliski.^13^ The MHCA has very stringent criteria for conditional discharge that few state patients fulfil. Thus state patients, most of them not returning from LOA, form a bigger proportion of our absconding population. This skews the figures and falsely gives the impression that dangerous forensic patients are escaping custodial care. The above finding differs substantially from that of Siwinska;^15^ the majority of the absconding samples were involuntarily detained general patients (i.e. Sections 9 and 12 of the recalled *Mental Health Act*, No. 18 of 1973).^10^ When an involuntarily admitted patient absconds, he is more likely to physically escape the hospital premises than a state patient (who is more likely not to return from LOA). Because of differences in legislation and detention of patients, it is difficult to compare South African studies with international studies.

The current study did not have a control/comparison group. If we extrapolate on the socio-demographic profile formulated in Mabena’s study on involuntary psychiatric admissions to two hospitals,^8^ a measure for potential comparison becomes possible. The mean age of the absconding group was 33.8 years. However, if only escaped patients were considered, the mean age was 26.2. This compares consistently with Siwinska’s findings of 35.5 and 29 years, respectively.^15^ Approximately 85% of the absconding sample was male. This reflects the greater number of male admissions (65.5% in Mabena’s study).^8^ These findings are also broadly consistent with other international findings.^5,6,18^ This profile is rather broad and vague and provides little guidance in addressing risk assessment and the prevention of absconding.

Only 22 patients escaped while under direct hospital supervision. The patients who escaped were more likely to be male, younger, involuntarily detained (under Section 34 of the MHCA)^10^ and admitted to a closed general ward. The possible reasons underlying this could include lack of physical impairment,^8^ efforts to obtain substances, feeling more restricted and poorer insight. They tended to escape within 6 months of admission and were more likely to report contributing factors prior to escaping. Fewer patients escaped from open wards, that is, 4 out of 22 (18.18%). This is in keeping with the theory by Khisty et al.,^19^ who hypothesised that the duration of stay in open wards is usually short and the environment less restrictive; therefore, the motivation for patients to escape from these wards is probably different.

The average length of a hospital stay in this study far surpasses international findings. Most studies do not include forensic psychiatric patients. Tomison (1989) cited in Khisty et al.^19^ found that a longer admission could be a predictor of absconding, especially in a closed-door, long-term facility.

Most of the studies from developed countries find that schizophrenia is the most common diagnosis in patients who abscond.^3,20^ This is corroborated by both the current study (45.36% of all absconding incidents) and Siwinska’s^15^ findings (up to 59%). Again, this could simply reflect an over-representation of this diagnosis in all patients admitted (corroborated by Janse van Rensburg^7^ and Mabena^8^). The second most common diagnoses are Primary Mood Disorder and Intellectual Deficit (19.59% each). The number of patients with intellectual deficit who were reported as having absconded is perhaps unexpected. These patients are usually signed out by responsible family members and the implication is that these family members did not think it necessary to return these patients on time.

The numbers of patients with a primary diagnosis of substance-induced disorders were not as high as in Siwinska’s study.^15^ This is not because of this condition being less frequent. It is likely that an underlying psychotic process is thought to exist in these patients and they are preferentially being diagnosed with a primary psychotic disorder. However, almost 75% of the absconding sample used substances of some kind, which is higher than Siwinska’s findings of 38%.^15^ A potential limitation is that in most files no differentiation was made between substance use and abuse. Janse van Rensburg,^7^ Mabena^8^ and Jonsson et al.^21^ indicate between 40% and 53% of patients fulfil criteria for substance abuse.

For the patients not returning from official LOA on time, reported factors contributing to the absconding included non-compliance, abusing substances, relapsing, re-offending with subsequent arrest, no money for transport, poor family support and adherence, suicide attempts, medical problems and attending cultural cleansing/initiation ceremonies.

For those escaping hospital custody, reported contributing factors included returning early from official LOA, LOA or discharge being denied, smoking cannabis, fighting with fellow patients, placed under constant special observations, no visitors, failed absconding attempts, pending placement and poor family support. In fact, a number of patients had voiced their intent to abscond prior to the actual incident. The reasons cited above are similar to those found by Siwinska,^15^ Bowers et al.^2^ and Bowers et al.^1^ These factors should be considered when drawing up a risk assessment tool.

Seven patients absconded repeatedly during the study period and were responsible for 18 absconding incidents. Falkowski et al.^20^ found that 39% of absconding incidents were repeat absconding incidents by the same individuals who absconded between 2 and 12 times. This makes one speculate as to whether any changes were made to the management of patients, once they returned.

Because of the retrospective nature of the methodology, the following limitations pertain: under-reporting of absconding incident, missing files and incomplete data leading to the small sample size. Because of time and resource constraints, a control population was not studied. Race, culture and ethnicity were not studied. In some instances, the diagnosis was preliminary and unclear. Substance abuse was not mentioned as a separate diagnosis. This does not provide accuracy with respect to the prevalence of dual diagnosis in the sample population.

Clear documentation was not always evident once patients had absconded or failed to return from LOA on time. This may have shed light as to their reasons for absconding and the circumstances surrounding their return. Most patients only re-established contact with psychiatric services once they had relapsed and been re-admitted. These limitations should not detract from the final results and conclusions of this study.

## Conclusion and implications for practice

The absconding rates in this study were lower than the international mean. These included patients not returning from official LOA. If these patients were excluded, then the absconding rate would have been even lower. The predominant method of absconding has also changed. A differentiation needs to be made between patients not returning from an official LOA and those physically escaping from hospital custody, as the risk assessment and management are very different. A socio-demographic profile of the potential absconder as described (i.e. single, unemployed male in their early 30s) is of limited value. It reflects the over-representation of these characteristics in admitted psychiatric patients.

The formulation and use of a risk assessment tool as a matter of routine on all admitted patients may be of great value. Patient, treatment and social factors should be considered. Patients deemed as high risk may warrant enhanced psychosocial and treatment interventions. The timeous and appropriate reclassification of state patients may reduce the population at risk of absconding.

Further research that builds on these South African findings would be useful to examine risk assessment and management practices in relation to absconding, directorate and hospital absconding policy and practices, as well as perceptions of mental healthcare users, staff and family members in relation to this stressful incident.
